# Comparison of Hematological Parameters Between First-Episode Schizophrenia and Anti-NMDAR Encephalitis

**DOI:** 10.3389/fcell.2022.895178

**Published:** 2022-07-06

**Authors:** Kai Huang, Yamei Tang, Zhiheng Chen, Shan Ding, Hongtao Zeng, Yuxu Zhao, Qi Yu, Yong Liu

**Affiliations:** ^1^ Department of Psychiatry and National Clinical Research Center for Mental Disorders, The Second Xiangya Hospital of Central South University, Changsha, China; ^2^ Department of Laboratory Medicine, The Second Xiangya Hospital, Central South University, Changsha, China; ^3^ Department of Pediatrics, The Third Xiangya Hospital, Central South University, Changsha, China; ^4^ School of Chinese Medicine, Hunan University of Chinese Medicine, Changsha, China

**Keywords:** first-episode schizophrenia, anti-NMDA encephalitis, NLR, SII, albumin

## Abstract

**Background:** First-episode schizophrenia (FES) and anti-NMDAR encephalitis are different disorders with similar psychiatric symptoms, and both diseases are associated with the inflammatory system. In this study, we compared hematological parameters and inflammation ratios in anti-NMDAR encephalitis, FES, and healthy control.

**Methods:** We enrolled 106 patients (53 FES patients and 53 anti-NMDAR encephalitis patients) and 59 healthy controls. The values of the neutrophil–lymphocyte ratio (NLR), platelet–lymphocyte ratio (PLR), monocyte–lymphocyte ratio (MLR), and systemic immune-inflammation index (SII) were used to evaluate inflammation. Other parameters such as the white blood cell (WBC), platelet (PLT), uric acid (UA), total bilirubin (TBIL), total bile acid (TBA), and serum albumin counts were also used to compare inflammation ratios between these two diseases.

**Results:** SII, NLR, PLR, MLR, and serum albumin levels were statistically significantly different between these three groups (*p* < 0.05). The values of SII, NLR, PLR, and MLR were significantly higher in the anti-NMDAR encephalitis group than those in the FES group (*p* < 0.05), and the values in both diseases were more increased than those in HC (*p* < 0.05). The serum albumin level was significantly lower in anti-NMDAR encephalitis than in FES (*p* < 0.05). WBC, neutrophil, lymphocyte, and monocyte counts showed significantly higher levels in the anti-NMDAR encephalitis group and FES group separately (*p* < 0.05). Other parameters like TBA, TBIL, and UA showed no difference between groups.

**Conclusion:** In summary, this is a relatively new study that is innovative by comparing some inflammation markers of peripheral blood in two diseases with clinically psychotic symptoms. These two diseases are related to the inflammatory system, proving that NMDAR dysfunction is related to psychotic symptoms. Besides, NLR, PLR, MLR, and serum albumin can be used as biomarkers to distinguish the two diseases. The serum albumin level in patients with anti-NMDAR encephalitis was lower than that in patients with schizophrenia.

## 1 Introduction

Anti-N-methyl- D-aspartate receptor (NMDAR) encephalitis is the most common autoimmune encephalitis, mediated by autoantibodies directly against the GluN1 subunit of the NMDAR (Gleichman et al., 2012). Patients with anti-NMDAR encephalitis often present with psychiatric symptoms, including memory loss, hallucinations, and paranoia, which are also seen in schizophrenia (SCZ). Furthermore, anti-NMDAR encephalitis is a type of autoimmune encephalitis that is closely related to inflammation, which is characterized by changes in local and systemic inflammatory parameters. Recently, in addition to white blood cells (WBC), platelets (PLT), neutrophils, lymphocytes, and monocytes, which are common inflammatory markers, the systemic immune-inflammation index (SII), the platelet–lymphocyte ratio (PLR), the neutrophil–lymphocyte ratio (NLR), and the monocyte–lymphocyte ratio (MLR), which are novel inflammatory markers, have been considered as useful indicators in infectious or inflammatory diseases (e.g., coronary artery diseases, transplant surgery, malignancies, and cancers) (Balkwill and Mantovani, 2010; Balta et al., 2016; Lolli et al., 2016; Tang et al., 2017; Chen et al., 2018a; Ohtaka et al., 2018; Zhan et al., 2018).

SCZ is a chronic mental disease with an onset typically in adolescence or young adulthood. Characteristic symptoms include hallucinations and delusions, as well as impairment of other cognitive abilities. The immune system processes in mediating the genetic and environmental risks for SCZ in animal and human studies (Drexhage et al., 2011; Upthegrove and Khandaker, 2019). Serologically suggested prenatal maternal infection with pathogens (such as influenza and herpes simplex virus 2) during pregnancy has been associated with SCZ (Khandaker et al., 2013). Individuals who have severe infections with a previous history of autoimmune diseases may have an increased risk of SCZ (Benros et al., 2011). Previous reviews have suggested links between SCZ and the immune system. Peripheral blood is the most direct and easiest way to indicate inflammation of one disease. Hematologic parameters, like leukocytes, PLT, neutrophils, lymphocytes, monocytes, SII, NLR, MLR, and PLR, are suitable for evaluating diseases related to the inflammatory system, which may indicate SCZ. NLR, MLR, and PLR increased quantitatively in mental disorders than in healthy individuals (Mazza et al., 2018; Mazza et al., 2020). In recent studies, PLT and lymphocyte counts have been significantly lower, while NLR and MLR counts have been significantly higher in SCZ patients than those in healthy individuals (Zhu et al., 2022).

Albumin is a metal-binding protein with the function of scavenging free radicals, which is considered an important antioxidant. The imbalance of antioxidants will produce free radicals, which will lead to a variety of inflammation-related diseases. Albumin is closely related to oxidative stress and antioxidant capacity (Lee et al., 2006; Chapple et al., 2007; Radhu et al., 2015; Causer et al., 2020; Oniki et al., 2020). Several studies have shown that serum albumin was significantly decreased in patients with SCZ (Babushkina et al., 2012; Chen et al., 2018b; Zhai et al., 2018). In addition, the serum albumin level is lower in anti-NMDAR encephalitis patients than that in healthy individuals (Jang et al., 2018; Shu et al., 2018). Bilirubin is also an antioxidant, which plays a role in protecting from oxidative stress (Kozaki et al., 1999; Yin et al., 2019). Similar to serum albumin, the total bilirubin (TBIL) level was found lower in SCZ patients than in healthy individuals (Reddy et al., 2003). Uric acid (UA) is an important free radical scavenger in the body, which can scavenge nitrogen peroxide free radicals and play an antioxidant role (Alvarez-Lario and Macarron-Vicente, 2011). A meta-analysis demonstrated that UA levels were decreased in FES subjects (He et al., 2020).

Both anti-NMDAR encephalitis and SCZ have similar psychiatric symptoms (Dalmau et al., 2011; Graus et al., 2016); it occurs if there might be a similar mechanism that causes psychiatric symptoms. However, few studies have assessed these parameters. In this study, we focused on the evidence of circulating inflammatory parameters and serological markers of inflammation. We aimed to establish the relationship between these two diseases and speculate on the potential mechanism of psychiatric symptoms.

## 2 Material and Methods

### 2.1 Study Design and Participants

This is a cross-sectional study of patients diagnosed with FES; there are many drugs and physiotherapies that may influence changes in inflammatory markers. We used FES as the study object of SCZ or anti-NMDAR encephalitis at The Second Xiangya Hospital of Central South University in 2018–2019. A total of 106 patients (53 patients with FES and 53 patients with anti-NMDAR encephalitis) were enrolled in the study. Furthermore, we also enrolled 59 healthy individuals from the Health Management Center of Second Xiangya Hospital in 2018–2019. WBC, neutrophil, lymphocyte, PLR, monocyte, serum albumin, uric acid, total bilirubin, and total bile acid levels were determined at the time of admission, and SII, NLR, PLR, and MLR were calculated. Sociodemographic data for these participants was also obtained from patient records.

SII, the systemic immune-inflammation index based on platelet (P), neutrophil (N), and lymphocyte (L) counts, was calculated using the formula P ×N/L; NLR was defined as N/L, MLR as monocytes (M)/L, and PLR as P/L at the baseline data.

### 2.2 Inclusion Criteria

The diagnosis of anti-NMDAR encephalitis needs all three of the following items checked (Graus et al., 2016): (i) at least four of the six major groups of symptoms occur within 3 months, including behavioral (psychiatric) abnormity or cognitive dysfunction, speech dysfunction, seizures, motor dysfunction, decreased level of consciousness, autonomic instability, or central hypoventilation; (ii) at least one of the following laboratory findings including EEG abnormity or CSF with pleocytosis or oligoclonal bands; (iii) exclude other disorders. In addition, all of the participants with anti-NMDAR encephalitis were having psychiatric symptoms, including positive or/and negative symptoms. The diagnosis of FES is based on the DSM-V, which is performed by at least two different psychiatric doctors, and the criteria are as follows: Two (or more) of the following, each present for a significant portion of time during a 1-month period: (i) delusions; (ii) hallucinations; (iii) disorganized speech; (iv) grossly disorganized or catatonic behavior; and (v) negative symptoms, that is, affective flattening, alogia, or avolition. All healthy group participants were in good physical health and without any abnormalities.

### 2.3 Exclusion Criteria

Patients with the following conditions were excluded: i) patients who took medicine before getting admitted to the hospital; ii) complicated with severe infections such as pneumonia and urinary tract infection or any other that will compromise patients’ inflammatory diseases; iii) complicated with systemic autoimmune diseases such as systemic lupus erythematosus, Kawasaki disease, and several types of tumors; iv) negative IgG anti-GluN1 antibodies present both in the serum and CSF; v) recent diagnosis of intracerebral hemorrhage or ischemic stroke; vi) missing data; and vii) all participants who do not have any chronic illness (infection diseases, hypertension, and diabetes mellitus) which could interfere the blood test and those do not take any medicines before diagnosis (steroids, antibiotics, etc.).

### 2.4 Statistical Analysis

All statistical analyses were performed using the software SPSS version 25.0. While *t*-test was used to assess normally distributed data, Mann–Whitney *U* test was used for non-parametric comparisons. One-way ANOVA tests were performed to establish the hematological parameters among data from different samples. Welch test was used when the homogeneity of variance was not ensured. For the pairwise comparison, LSD test was used in the case that the variances are homogenous and Games-Howell test was used when the variances are not homogenous. Average and standard deviation values were given as descriptive statistics. *p* < 0.05 was accepted as statistically significant.

## 3 Results

### 3.1 Baseline Patient Characteristics

A total of 106 patients, including 53 FES patients and 53 anti-NMDAR encephalitis patients, and 59 healthy controls (HCs) were enrolled. The characteristics of these groups are shown in Table 1. In terms of gender, there were no differences among these groups. However, there were significant differences in mean ages (30.91 ± 13.38 years, 22.04 ± 8.70 years, and 35.56 ± 10.33 years, respectively) (Welch = 29.068; *p* < 0.05).

**TABLE 1:** Characteristics of participants with anti-NMDAR encephalitis, first-episode schizophrenia, and healthy individuals.

	Anti-NMDAR encephalitis (n = 53)	FES (n = 53)	Control (n = 59)	X2/Welch	*p-*value
Age (years)	30.91 ± 13.38	22.04 ± 8.70	35.56 ± 10.33	29.068	**0.000**
Gender	-	-	-	0.054	0.973
Male	28	27	30	-	-
Female	25	26	29	-	-

Data were expressed as the means ± standard deviation (SD) for the Welch test and others calculated by chi-square. Bold values are statistically significant findings (*p* < 0.05). FES, First-episode schizophrenia; Control, control group (healthy individual).

### 3.2 Comparison of Hematological Parameters Among Groups

Mean values of hematological parameters are given in Table 2. The values include the levels of WBC, PLT, neutrophils, lymphocytes, eosinophils, monocytes, SII, NLR, PLR, MLR, serum albumin, UA, TBIL, and TBA. All the aforementioned groups showed significant differences among them in addition to UA, TBIL, and TBA groups.

**TABLE 2:** Comparison of factors in anti-NMDAR encephalitis, FES, and healthy individuals.

	Anti-NMDAR encephalitis (n = 53)	FES (n = 53)	Control (n = 59)	Welch test/F	*p-*value
WBC, ×10^9^/L	8.99 ± 2.95	6.81 ± 2.22	6.33 ± 1.24	18.224	**0.000**
PLT, ×10^9^/L	264.13 ± 74.80	242.47 ± 52.17	229.64 ± 53.40	3.863	**0.000**
Neutrophils, ×10^9^/L	6.92 ± 3.28	4.47 ± 2.13	3.82 ± 0.99	22.343	**0.000**
Lymphocytes, ×10^9^/L	1.60 ± 0.47	1.95 ± 0.69	2.03 ± 0.45	12.348	**0.000**
Eosinophils, ×10^9^/L	0.08 ± 0.09	0.09 ± 0.06	0.16 ± 0.09	15.297	**0.000**
Monocytes, ×10^9^/L	0.49 ± 0.19	0.42 ± 0.23	0.31 ± 0.08	24.294	**0.000**
SII	1,049.83 ± 600.21	537.42 ± 261.29	436.44 ± 163.84	24.157	**0.000**
NLR	4.71 ± 3.69	2.57 ± 1.46	1.94 ± 0.59	90.516	**0.000**
PLR	171.59 ± 80.22	134.16 ± 41.01	116.49 ± 30.96	12.296	**0.000**
MLR	0.34 ± 0.23	0.23 ± 0.14	0.16 ± 0.06	17.563	**0.000**
Albumin, g/L	40.00 ± 5.19	43.00 ± 6.06	46.10 ± 2.66	31.736	**0.000**
UA, umol/L	304.3 ± 116.36	340.61 ± 95.37	318.05 ± 87.22	1.735	0.180
TBIL, umol/L	10.39 ± 4.29	12.72 ± 7.04	10.52 ± 4.58	2.272	0.108
TBA, umol/L	3.44 ± 2.47	4.25 ± 2.51	3.31 ± 2.05	2.411	0.093

The Welch test was used in this table because the data did not meet the basic requirements of the *t*-test. Others used ANOVA because it cannot match the homogeneity of variances. Some of the data have been stripped of extreme values. WBC, white blood cell; PLT, platelet; SII, immune-inflammation index; NLR, neutrophil–lymphocyte ratio; PLR, platelet–lymphocyte ratio; MLR, monocyte–lymphocyte ratio; UA, uric acid; TBIL, total bilirubin; TBA, total bile acid. Bold values are statistically significant findings (*p* < 0.05).

**FIGURE 1:**
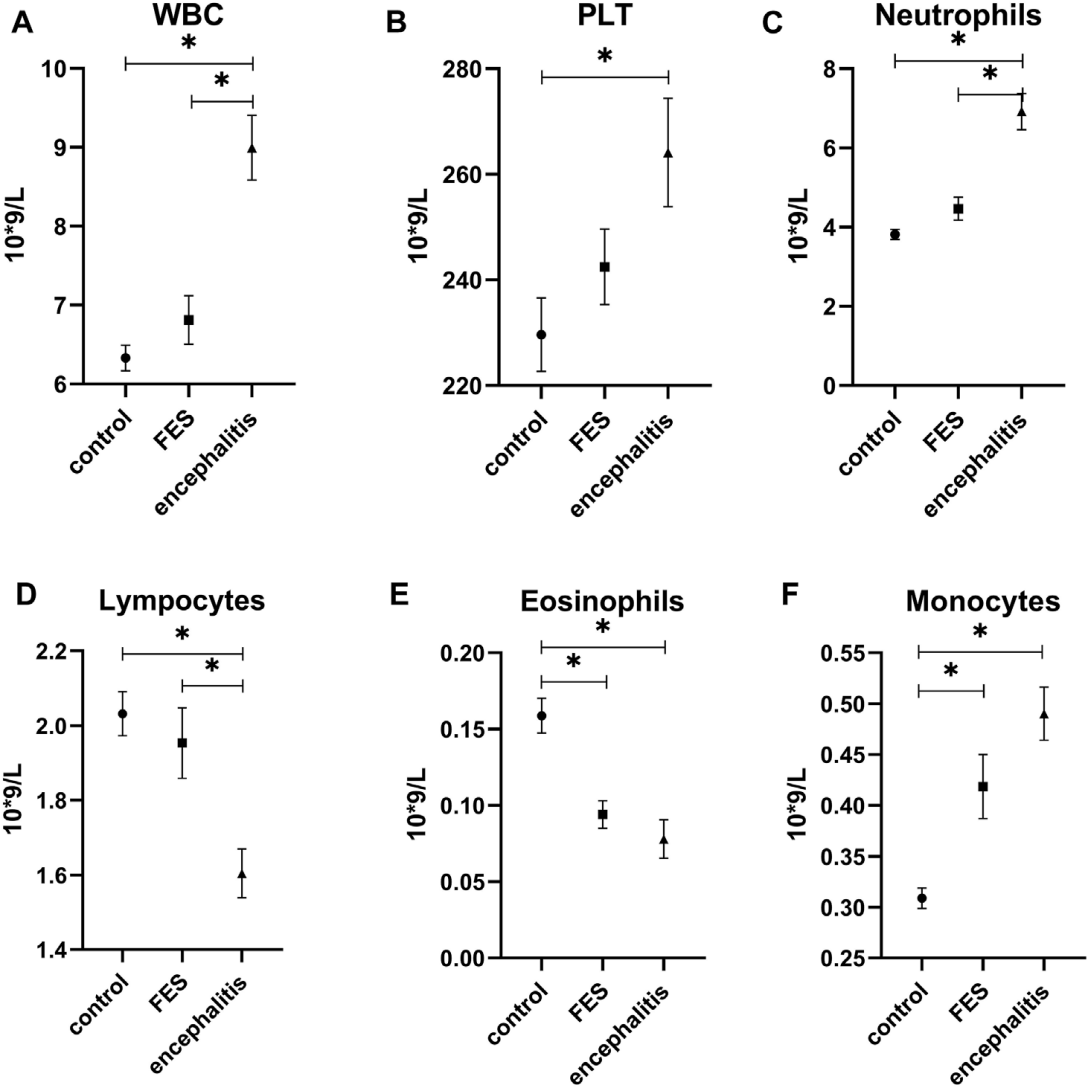
Comparison of WBC, PLT, neutrophils, lymphocytes, and eosinophils counts in anti-NMDAR encephalitis, FES, and healthy control. FES, first-episode schizophrenia; encephalitis, anti-NMDAR encephalitis; WBC, white blood cell; PLT, platelet. * *p* < 0.05. All the above hematological indicators except eosinophils were used Games-Howell tests, and the other two used the LSD test.

**FIGURE 2:**
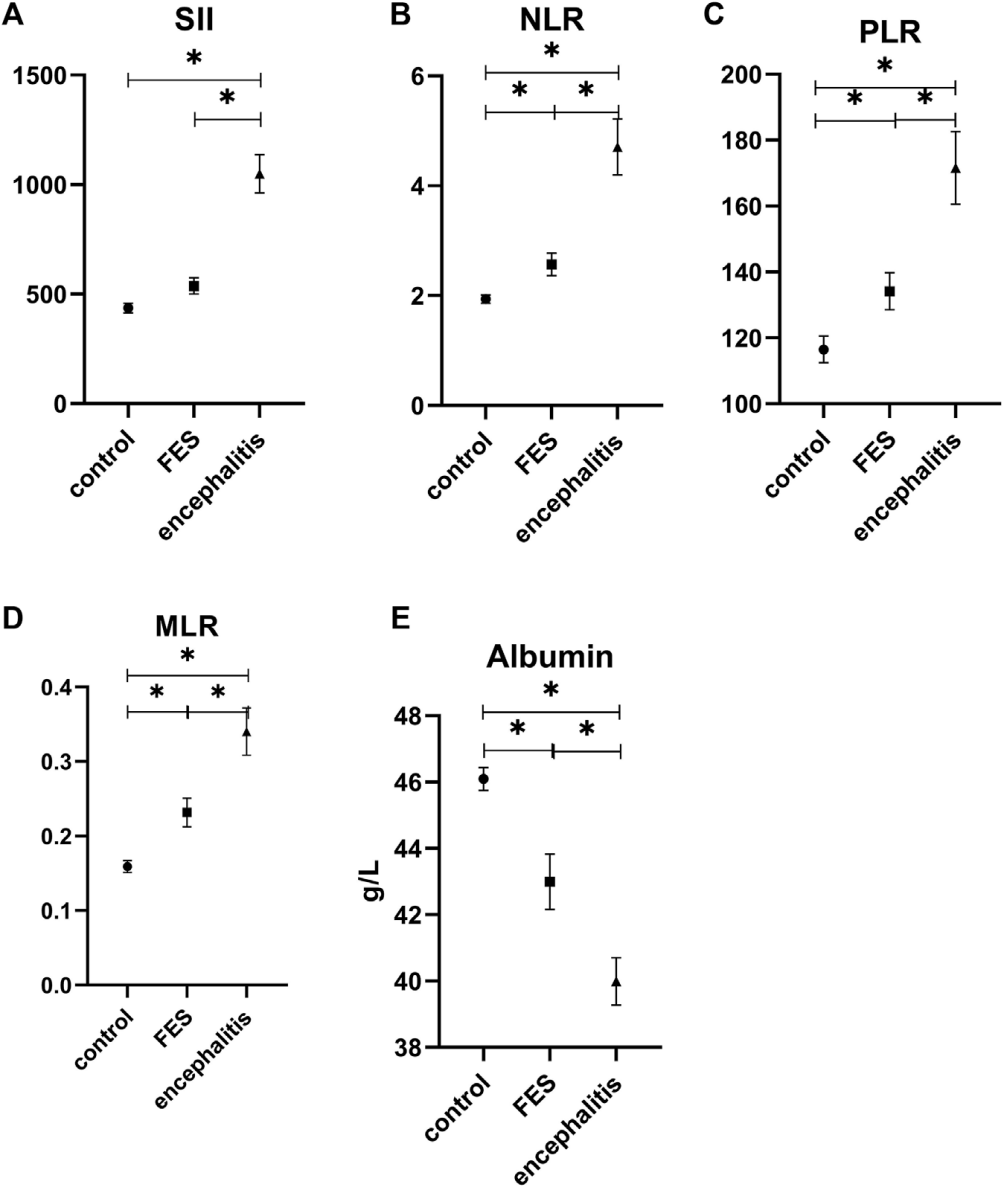
Comparison of monocytes counts, SII, NLR, PLR, MLR, and serum albumin level in anti-NMDAR encephalitis, FES, and healthy control. FES, first-episode schizophrenia; encephalitis, anti-NMDAR encephalitis; SII, immune-inflammation index; NLR, Neutrophil Lymphocyte Ratio; PLR, Platelet Lymphocyte Ratio; MLR, Monocyte Lymphocyte Ratio. * *p* < 0.05. All the above parameters were using Games-Howell tests.

In the multiple comparison analysis (Figures 1, 2.), the WBC count in patients with anti-NMDAR encephalitis (8.99 ± 2.95×109/L) is significantly higher than that in the FES group (6.81 ± 2.22×109/L; *p* = 0.000) and HCs (6.33 ± 1.24×109/L; *p* = 0.000), and the PLT count in patients with anti-NMDAR encephalitis (264.13 ± 74.80×109/L) is significantly higher than that in the HCs (229.64 ± 53.40×109/L; *p* = 0.000). Also, the level of neutrophils showed the same as WBC (6.92 ± 3.28×109/L, 4.47 ± 2.13×109/L, and 3.82 ± 0.99×109/L; *p* = 0.000 and *p* = 0.000). However, lymphocytes in the anti-NMDAR encephalitis group (1.60 ± 0.47×109/L) were significantly lower than those in the FES group (1.95 ± 0.69×109/L; *p* = 0.008) and HCs (2.03 ± 0.45×109/L; *p* = 0.000). In terms of eosinophil count, the anti-NMDAR encephalitis group (0.08 ± 0.09×109/L) was lower than the HCs (0.16 ± 0.09×109/L; *p* = 0.000), and the FES group (0.09 ± 0.06×109/L) was also lower than the HCs (*p* = 0.000). On the contrary, monocytes in the anti-NMDAR encephalitis group (0.49 ± 0.19×109/L) were significantly higher than those in the HCs (0.31 ± 0.08×109/L; *p* = 0.000) and FES group (0.42 ± 0.23×109/L) and is also higher than the HCs (*p* = 0.004). SII in the anti-NMDAR encephalitis group (1049.83 ± 600.21) was significantly higher than that in the FES group (537.42 ± 261.29; *p* = 0.000) and HCs (436.44 ± 163.84; *p* = 0.000). The levels of NLR, PLR, MLR, and serum albumin showed a significant difference between each group. In the levels of NLR, PLR, and MLR, the anti-NMDAR encephalitis group (respectively, 4.71 ± 3.69, 171.59 ± 80.22, and 0.34 ± 0.23) was significantly higher than the FES group (respectively, 2.57 ± 1.46, 134.16 ± 41.01, and 0.23 ± 0.14; *p* = 0.000, *p* = 0.002, and *p* = 0.010) and HCs (respectively, 1.94 ± 0.59, 116.49 ± 30.96, and 0.16 ± 0.06; *p* = 0.000, *p* = 0.002, and *p* = 0.000), and the FES group (respectively, 2.57 ± 1.46, 134.16 ± 41.01, and 0.23 ± 0.14) was also significantly higher than HCs (respectively, *p* = 0.014, *p* = 0.002, and *p* = 0.002). The level of serum albumin in the anti-NMDAR encephalitis group (40.00 ± 5.19) was significantly lower than that in the FES group (43.00 ± 6.06; *p* = 0.020) and HCs (46.10 ± 2.66; *p* = 0.000), and the FES group was also significantly lower than the HCs (*p* = 0.003).

## 4 Discussion

Psychiatric symptoms are similar in both anti-NMDAR encephalitis and SCZ diseases. Recently, inflammatory scales have been proved significantly associated with SCZ in numerous studies (Drexhage et al., 2011; Mazza et al., 2018; Upthegrove and Khandaker, 2019; Mazza et al., 2020). In this study, we have shown that inflammation scales have a close relationship with FES and anti-NMDAR encephalitis. SII, NLR, PLR, and MLR levels were significantly increased in the anti-NMDAR encephalitis group compared to the FES group. Antioxidant substance and serum albumin levels were significantly lower in the anti-NMDAR encephalitis group than those in the FES group.

Anti-NMDAR encephalitis is an inflammatory disorder that disrupts the brain, causing psychiatric symptoms and neurological dysfunction. The abnormal immune response triggered by inflammation induced by the disease leads to autoimmune disease progression (Zeng et al., 2019). As an autoimmune inflammatory disease of the central nervous system, its pathogenesis has not been fully elucidated, but it has been proved to be related to the inflammatory response. The innate immune system is an early body defense mechanism that is the first to recognize and attack microbes or toxins that enter the organism. Immune cells are recruited to the areas of infection or inflammation by producing various chemicals, including cytokines. At the same time, activated antigen-presenting cells and T lymphocytes activate the adaptive immune response, promoting the proliferation, differentiation, and maturation of different lymphocyte subsets and play a crucial role in the pathogenesis of autoimmune encephalitis (Weissert, 2017). Hence, as a systemic inflammatory index, SII, NLR, PLR, and MLR could be the biomarkers associated with inflammation secondary to brain injury. Anti-NMDAR encephalitis and SCZ both have abnormal behavior (psychosis, delusions, hallucinations, agitation, aggression, or catatonia) and cognitive dysfunction (Dalmau et al., 2011; Graus et al., 2016). In recent years, SCZ also has been confirmed to be related to inflammation, which includes circulating cytokines, oxidative stress, and cellular markers (Müller, 2018; Upthegrove and Khandaker, 2019). NLR, MLR, and PLR have already been investigated in psychological disorders, and most of them showed an increase in ratio compared to healthy individuals (Mazza et al., 2018; Mazza et al., 2020). Özdin and Böke (2019)reported that the early onset of positive symptoms in SCZ patients showed lower lymphocytes and higher neutrophils. Our study found that NLR, PLR, and MLR were significantly higher in the FES group than in the HCs, which is consistent with previous studies (Özdin et al., 2017; Mazza et al., 2018; Mazza et al., 2020). Moreover, SII, NLR, PLR, and MLR were higher in the anti-NMDAR encephalitis group and FES group than in HCs. Also, NLR, PLR, and MLR in the anti-NMDAR encephalitis group were higher than those in the FES group. Both diseases are associated with inflammation, while the anti-NMDAR group is associated with a remarkable rise. It is recommended that both diseases are related to inflammation, especially in the anti-NMDAR group. In conclusion, the inflammatory response is better in the anti-NMDAR encephalitis group than in the FES group, especially LRL, PLR, and MLR.

Glutamate overactivation of the postsynaptic NMDAR may lead to neuronal damage (Zhang et al., 2019). Studies have shown that oxidative stress results in over-activation of NMDA and altered dopamine receptor function in psychosis (Upthegrove and Khandaker, 2019). Nevertheless, NMDAR antagonists have been used to stimulate positive and negative symptoms and cognitive deficits in SCZ by blocking glutamate-mediated excitatory neurotransmission (Radhu et al., 2015). Previous animal and human studies have shown that the NMDAR hypofunction leads to these symptoms. Unfortunately, no IgG NMDAR antibodies were identified in SCZ patients in previous studies (Hara et al., 2018). It seems that both diseases are related to NMDAR and have been closely related to NMDAR dysfunction and psychiatric symptoms. In our study, the inflammatory index was significantly lower in the anti-NMDAR encephalitis group than that in the FES group, which may explain why it cannot detect NMDAR antibodies in the FES. Even though the numbers are low, we cannot deny the existence of similar mechanisms. Although inflammation values indicate a similar trend in anti-NMDAR and FES disease, especially NLR, PLR, and MLR, changes were not similar. One possible reason is that SCZ may be an inflammatory response to stress, while encephalitis is an autoimmune inflammation caused by antibodies, so inflammatory markers may not perform as well as encephalitis. Another reason for this kind of situation is that the mechanism of SCZ is far more complex than that of anti-NMDAR encephalitis. For instance, disturbance of dopaminergic neurotransmission in SCZ may interfere with the inflammatory cytokines. In addition, SCZ animal experiments have shown that disrupted-in-schizophrenia-1 (DISC1) influences the expression and function of NMDAR through phosphodiesterase 4/protein kinase A/cyclic adenosine monophosphate response element-binding protein (CREB)–dependent mechanisms, thus affecting NMDAR-dependent cognition and mood situations (Wei et al., 2014). Other than this, the cystine/glutamate antiporter system xc− is related to glutamate-release regulation. SCZ patients exhibited downregulation of xc− subunits, especially the solute carrier (SLC) family (Lin et al., 2016). System xc− plays a critical role in glutamate release, which may connect to hypoglutamateric hypotheses of SCZ. The mRNA levels of some system xc− subunits are lower in SCZ patients (Hung et al., 2021). In brief, NMDA receptor dysfunction is not the only reason that causes hypoglutamateric hypothesis; other mechanisms contribute to this; therefore, this is one of the reasons we cannot detect IgG anti-GluN1 antibodies.

Albumin is a metal-binding protein with free radical scavenging properties. It is also considered an important antioxidant, which plays a protective role in the inflammatory response. Studies have shown oxidative stress-driven interneuron impairment in models of SCZ (Steullet et al., 2017). In our study, albumin levels were significantly reduced in both diseases, which are consistent with previous studies (Chen et al., 2018b; Jang et al., 2018; Shu et al., 2018). In SCZ, oxidative stress may be caused by dysfunctions that typically affect the system, including glutamate, dopaminergic, immune, and antioxidant signaling. In addition, other researchers have performed relevant studies and found that serum albumin levels in patients with anti-NMDAR encephalitis significantly increased after immunotherapy, which further proves that albumin may be a protective indicator (Jang et al., 2018). In our study, the serum albumin level was lower in patients with anti-NMDAR encephalitis than in FES. As a protective factor, albumin was significantly reduced in these two diseases compared with the healthy controls and more in anti-NMDAR disease, suggesting that this antioxidant effect is crucial for inflammation-mediated diseases, which further indicated that the influence of inflammation on these two diseases is the basis of disease. In other words, serum albumin may be significantly associated with psychiatric symptoms, and the changes in anti-NMDAR encephalitis patients are more obvious than those in FES patients. Although serum albumin in both diseases was lower than in healthy controls, that in anti-NMDAR encephalitis was lower than in FES. It could be some gene mutations that induce these conditions. For example, NRGs, ErbB4, α7nAChR, and SR are many susceptibility genes known to be linked to NMDAR-mediated glutamatergic neurotransmission in SCZ (Pocklington et al., 2014).

## 5 Limitation

It should be noted that there are still several limitations to our study. First, our sample size is relatively small. Second, this was a cross-sectional study that does not allow the determination of cause-and-effect associations. Last, there was no scale to evaluate the psychiatric symptoms.

## 6 Conclusion

In summary, this is a relatively new innovative study comparing the blood inflammatory markers of two diseases with similar clinical psychiatric symptoms. These two diseases showed the same trend of increasing inflammatory indicators, but the magnitude of change between these two diseases was statistically significant. The authors believe that both diseases are related to hypofunction NMDAR, but the mechanisms of SCZ seem more complex than those of anti-NMDAR encephalitis. We know that both diseases relate to the inflammatory system and oxidative stress, which may prove the NMDAR hypofunction related to psychiatric symptoms. This study not only proves SCZ is associated with the immune system but also suggests that there are differences between these two diseases in inflammatory markers. In the future, they may become new indicators for discriminating between the two diseases.

## Data Availability

The raw data supporting the conclusion of this article will be made available by the authors, without undue reservation.
